# The Outer Vestibule of the Na^+^ Channel–Toxin Receptor and Modulator of Permeation as Well as Gating

**DOI:** 10.3390/md8041373

**Published:** 2010-04-21

**Authors:** René Cervenka, Touran Zarrabi, Peter Lukacs, Hannes Todt

**Affiliations:** Institute of Pharmacology, Centre of Physiology and Pharmacology, Medical University of Vienna, 1090 Vienna, Austria; E-Mails: rene.cervenka@meduniwien.ac.at (R.C.); touranzar@yahoo.com (T.Z.); peter.lukacs@meduniwien.ac.at (P.L.)

**Keywords:** tetrodotoxin, saxitoxin, sodium channel, use-dependent block, rate-dependent block, outer vestibule

## Abstract

The outer vestibule of voltage-gated Na^+^ channels is formed by extracellular loops connecting the S5 and S6 segments of all four domains (“P-loops”), which fold back into the membrane. Classically, this structure has been implicated in the control of ion permeation and in toxin blockage. However, conformational changes of the outer vestibule may also result in alterations in gating, as suggested by several P-loop mutations that gave rise to gating changes. Moreover, partial pore block by mutated toxins may reverse gating changes induced by mutations. Therefore, toxins that bind to the outer vestibule can be used to modulate channel gating.

## 1. Introduction

**Figure 1:**
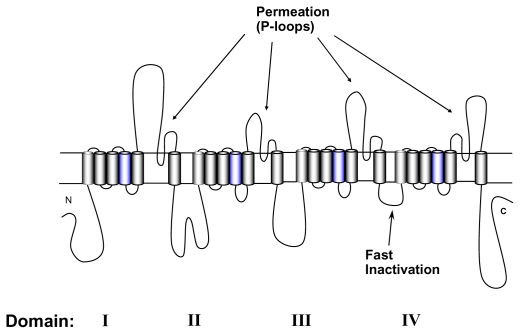
Transmembrane-folding diagram of the α-subunit of the voltage-gated Na^+^ channel. Probable α-helical segments and polypeptide chains are represented as cylinders and bold lines, respectively. The bold lines represent polypeptide chains. S4 segments (blue) are the voltage sensors.

Voltage-gated Na^+^ channels permit rapid transmission of depolarizing impulses throughout cells and cell networks, which forms the basis of function of skeletal muscle, the heart and the nervous system. They consist of one pore forming α subunit, and of up to two accessory β subunits [1,2]. Heterologous expression of α subunits alone is sufficient to form functional Na^+^ channels [3,4]. The α-subunit is a large polypeptide of about 1,800 amino acids containing four repeated domains of 300 to 400 amino acids. Each domain (DI-DIV) consists of six membrane spanning α-helical segments (Figure 1). The S4 segments in each domain contain positively charged amino acid residues at every third position. These residues serve as voltage sensors thereby controlling the gating machinery of the channel [5]. The short intracellular loop connecting homologous domains III and IV serves as the inactivation gate, blocking the pore from the inside during sustained depolarization of the membrane. The outer vestibule is generally considered to be composed of the external loops connecting S5 and S6 segments. These loops fold into the channel pore and form the selectivity filter at the innermost turn [6–8]. Tetrodotoxin (TTX) and Saxitoxin (STX) are naturally occurring guanidinium toxins that bind strongly to the outer vestibule of voltage-gated Na^+^ channels thereby occluding the permeation pathway (for review see [9]). The binding site has been mapped to a region immediately external to the selectivity filter [10–14].

The outer vestibule has long been considered a rigid structure to which TTX binds without involvement of substantial conformational changes which would result in the modification of gating [15–17]. Contrary to this view, studies in which spontaneous or catalyzed crossliniking of double cysteine mutants in the outer vestibule was used as a readout for conformational changes suggested that the P-loops are highly flexible structures and that conformational changes in this region may be linked to gating transitions [18,19]. Such conformational flexibility may be important for toxin–channel interactions. This has been suggested using computational methods [20,21] and was confirmed by Lange and colleagues [22]: Using high-resolution solid-state NMR spectroscopy they demonstrated that high-affinity binding of the scorpion toxin kaliotoxin to a chimaeric K^+^ channel was associated with significant structural rearrangements in both molecules. Conformational changes of the outer vestibule of voltage-gated Na^+^ channels may also result from binding of small molecules to their receptor site [23]. Thus, it is reasonable to assume that binding of TTX may alter the channel conformation. If such conformational change was linked to certain gating transitions this could result in use-dependent block, similar as has been reported for the interactions of local anesthetic drugs with voltage-gated Na^+^ channels [24–27]. As a matter of fact, there are a number of reports of such use-dependent block of voltage-gated Na^+^ channels by TTX and STX. In the following section the development of the concepts of use-dependent block by guanidinium toxins will be reviewed.

## 2. Use-dependent Block by Guanidinium Toxins

Nine mammalian voltage-gated Na^+^ channel isoforms have been identified (Na_V_1.1-Na_V_1.9) and functionally expressed. They are all greater than 50% identical in amino acid sequence in the transmembrane and extracellular domains. In addition, atypical sodium channel-like proteins have been found in several organs but these have not been functionally expressed (Na_x_) [6]. The neuronal channels Na_V_1.1, Na_V_1.2, Na_V_1.3, Na_V_1.6, Na_V_1.7 and the skeletal muscle channel Na_V_1.4 are TTX-sensitive (EC_50_ in the nanomolar range). The neuronal channels Na_V_1.8, Na_V_1.9 and the cardiac channel Na_V_1.5 are TTX-resistant (EC_50_ in the micromolar range). The resistance to TTX is conferred by a change in amino acid sequence at a single position in the P-loop of domain I (see [9] for review). The first studies showing voltage-dependent or use-dependent block by TTX were performed on TTX-resistant channels.

In 1976 Baer [28] *et al.* showed in TTX-insensitive guinea pig papillary muscles that a depolarization of the resting potential from −89 mV to −71 mV increased the TTX affinity by a factor of about 20. In that study the maximal upstroke velocity (V_max_) of the cardiac action potential was used as an indirect and nonlinear measure of the Na^+^ conductance. The authors concluded that the voltage-dependent effects of TTX indicated a basic difference “in the chemistry or configuration of the TTX receptor”.

In 1981 Cohen *et al.* [29] performed a systematic investigation of Na^+^ currents in TTX-insensitive rabbit Purkinje fibres using the voltage-clamp technique. They described the basic features of use-dependent block by TTX namely an additional reduction in Na^+^ currents during repetitive pulses and the appearance of an additional fraction of slowly recovering channels after a single depolarizing pulse. In their study TTX did not change the voltage-dependence of I_Na_ availability after prolonged conditioning pulses (15–18 s). Cohen *et al.* explained their data by assuming binding of TTX to closed, open, and two inactivated states. In their scheme TTX binds to these states with equal affinity but with different rates.

Gonoi *et al.* [30] identified TTX-sensitive as well as TTX-insensitive populations of Na^+^ channels in cultured rat skeletal muscle cells converted to myoballs by treatment with colchicine. The cells were stimulated with depolarizing test pulses to −15 mV for 10 ms at a frequency of 2 Hz. The authors found frequency-dependent TTX block only in TTX-insensitive channels but not in TTX-sensitive channels.

Vassiliev *et al.* [31] measured single Na^+^ channel currents in isolated guinea pig ventricular myocytes using the patch clamp technique. Their technique to assess ensemble average Na^+^ currents had the advantage of allowing the properties of myocardial currents to be studied under physiological conditions (normal external Na^+^ concentrations) and in the absence of a significant series resistance as encountered in whole-cell Na^+^ current measurements. However, this technique is limited by the inability to compare conditions in the presence and absence of the drug in the same patch. 0.5 μM and 1 μM TTX produced −10 and −17 mV shifts of the availability curve, respectively. These results contrast with the findings by Cohen *et al.* [29] in rabbit Purkinje fibres where TTX did not shift the I_Na_ availability curve.

Salgado *et al.* [32] investigated the use- and voltage-dependent effects of STX in crayfish giant axons. The authors studied the effects of STX whereas only use-dependent effect of TTX had been reported previously. The use of STX was justified with the much faster dissociation rate of STX *versus* TTX. Also these authors investigated the use-dependent effects in TTX-sensitive currents, using nanomolar toxin concentrations, whereas previous work was done on TTX-resistant currents.

STX had a frequency-dependent blocking effect with the amount of block increasing with increasing frequencies. Short prepulses (few ms) induced a delayed development of block after the pulse. Thus, during hyperpolarization following the conditioning pulse the current first declined during approximately 10 s and then recovered over a prolonged period of 50–100 s. Makielski *et al.* later referred to this phenomenon as “post-repolarization block” [33]. Examination of the development of this block by varying the prepulse duration indicated full development of block during 1 ms. This indicated that the extra block did not require inactivation because at 1 ms very few channels would inactivate. Furthermore, the authors demonstrated that use-dependent block during rapid repetitive stimulation increased at more negative holding potentials.

The authors explained their data using a model in which a Na^+^ ion occupying the pore electrostatically repels the toxin molecule. In the resting state the toxin would still bind to the outer mouth but the repulsion results in a low affinity block in which the ion is trapped by the toxin within the channel. Upon depolarization the activation gate opens thereby allowing the trapped ion to escape into the cytoplasm. Upon repolarization the toxin unbinds from ion-depleted channels. At the same time toxin molecules start to bind to closed channels occupied by ions. If the latter process is faster than the former total block will temporarily increase (fast binding to resting channels) and then recover (slow unbinding from ion-depleted channels).

Carmeliet studied the effect of TTX on slowly inactivating Na^+^ currents in rabbit cardiac Purkinje fibers at 37 °C [34]. Micromolar concentrations of TTX speeded the decay of the currents and left-shifted the availability curve by 10 mV indicating preferential binding to inactivated states. The author suggested the discrepancy of this result with the absence of a toxin-induced shift in the availability curve in the earlier study by Cohen *et al.* [29] may be explained by the different nature of the investigated inactivated states *i.e.* slow inactivation in his study *versus* fast inactivation in the study by Cohen.

Clarkson *et al.* investigated possible state-dependent blocking activity of TTX in guinea pig papillary muscle at 37 °C [35]. Using V_max_ of the action potential upstroke as indicator of Na^+^ conductance the authors investigated possible state-dependent effects of TTX on guinea pig papillary muscles. During superfusion with micromolar concentrations of TTX these authors found rate-dependent inhibition of V_max_ during high frequency repetitive pulsing and a substantial increase in the time constant of recovery from block induced by repetitive pulsing. TTX induced a −5 mV shift of the availability curve for fast inactivation. The data could be well fitted by a modulated receptor model by assuming high affinity binding to inactivated channels.

Lönnendonker [36] was first to compare TTX and STX with regard to use-dependent action. Using voltage-clamp in myelinated frog nerve fibers he found that use-dependent block by rapid train pulses with nanomolar concentrations of TTX and STX was increased at negative holding potentials. Negative holding potentials increased the fraction of blocked current but not the time course of block development. Following a single short depolarization extra block developed at hyperpolarized potentials followed by slow recovery, a phenomenon later referred to as post-repolarization block [33]. The development of this block was accelerated with higher toxin concentrations. Also, STX had a faster time course of block development and recovery than TTX. During depolarization, block development was complete after 0.2 ms and did not change over a period of 12 ms indicating that the affinity change of the toxin receptor was triggered by a rapid activation whereas subsequent inactivation did not result in further modification. It has to be noted that their experiments were conducted at 15 °C such that inactivation would be expected to be delayed. The reported effects were described quantitatively by assuming a fast increase in receptor availability during channel activation followed by slow toxin binding and relaxation of the receptor affinity. Within the framework of their model STX exhibited shorter on-rates and off-rates than TTX.

In 1990 Eickhorn *et al.* [37] investigated the use dependent block of Na^+^ currents in rat cardiac myocytes. 5.5 μM TTX shifted the availability curve determined with 10 s conditioning prepulses by 17 mV to the left, without altering the slope of the Boltzmann relationship. However, with prepulses of shorter duration (250 ms) TTX did not shift the availability curve. The authors concluded that TTX binds to the inactivated state without strong interaction between drug charge and the membrane field. They also assume binding to a second inactivated state with very slow binding kinetics.

Frequency-dependent block developed at 1 Hz and saturated at 10 Hz. This behaviour was well fitted by a guarded receptor model [25] assuming equal affinities to pre-activated, activated and inactivated channels.

Patton and Goldin [38] investigated the state-dependence of TTX block using mutants of rNa_V_1.2 channels in which activation and inactivation gating was altered. A construct in which nine amino acids were inserted at the end of domain III S6 and three amino acids were inserted at the beginning of domain IV S6 had a 2-fold slower macroscopic rate of inactivation without altering the voltage dependence of activation. In this mutant the kinetics of use-dependent extra block by TTX was similar to wild-type suggesting that use-dependent block was not dependent on entry into the fast-inactivated state. On the other hand, in the mutant L860F, which shifted the voltage-dependence of activation by 20 mV into the depolarizing direction, the voltage-dependence of extra block was also shifted by a similar amount to the same direction. Thus, the conformational change related to high affinity block was likely to be related to activation. However, both in wild-type and in L860F the voltage-dependence of extra-block was more negative than the voltage dependence of activation suggesting that the conformational change related to high-affinity binding occurs during a closed-state gating transition that takes place at potentials more negative than activation.

Lönnendonker [39] investigated use-dependent block with TTX and STX at frog Ranvier nodes. He found that use-dependent block during trains of depolarizing pulses was more pronounced at negative holding potentials. Furthermore the voltage-sensitivity of use-dependent block was greater with STX than with TTX, which this author explained by the higher charge and the faster binding kinetics. Also, the step voltages of the test pulses eliciting use-dependent block were in the same range as the voltage dependence of steady state inactivation suggesting that inactivation enhances the affinity for toxin binding.

This author also explored the effect of external cations on use-dependent block in myelinated frog nerve fibres [40]. Increasing the Ca^2+^ concentration shifted the voltage dependence of use-dependence. Lowering external Ca^2+^ to 0.2 mM abolished use-dependence, even if the external Na^+^ concentration was doubled. External Mg^2+^ decreased use-dependence while external La^3+^ increased use-dependence. The author proposed that divalent or trivalent cations bind to a deep site in the channel thereby reducing TTX or STX binding to an external receptor. However, at higher divalent cation concentrations with La^3+^ in the external solution, the time constants of development of use-dependence increased over the values of the on-time constant of toxin block in Ringer solution. This indicated that the number of free binding sites increases slowly during repetitive pulsing.

Makielski *et al.* [33] investigated the properties of phasic block by STX in Na^+^ currents of ventricular myocytes from rat hearts. With 50 nM STX recovery from a 5 s depolarizing prepulse was biexponential with a time constant of 0.31 s presumably reflecting slow recovery from inactivation and a second time constant of 4.9 s, not seen under control conditions, presumably reflecting recovery of STX blocked channels. Short conditioning pulses (10 ms) elicited a different time course: for short recovery times (50 ms) I_Na_ was not blocked. Thereafter, a block developed which then recovered at longer interpulse intervals. Such “post-repolarization block” had been previously reported with STX and TTX in non-cardiac preparations [32,38,36]. Only short pulses (~10 ms) elicited post-repolarization block. In order to account for the biphasic time course of block during recovery, the authors developed a three state model in which STX binds to a transient closed state which is traversed during depolarization and repolarization.

Residue C374 in the outer vestibule of rNa_V_1.5 channels is a critical determinant for TTX resistance [41]. If mutated to the neuronal-specific amino acid phenylalanine channels acquire a high sensitivity for STX block, similar to wild-type neuronal channels. Satin *et al.* showed that the C374F mutation also confers the isoform-specific properties of post-repolarization block by STX [42]. Thus, both the development and the recovery of post-repolarization block by STX were substantially slower in rNa_V_1.2 and rNa_V_1.5 C374F channels than in wild type rNa_V_1.5 channels. Application of a three-state kinetic model [33] predicted a faster toxin dissociation rate, slower association rate, and shorter dwell time in a putative high STX affinity conformation for the cardiac isoform. The isoform-dependent differences in dwell times for the high affinity state imply that the amino acid that controls affinity to guanidinium toxins may also modulate gating behavior. Hence, the amino acid at position 374 in rNa_V_1.5 and in the analogous position in rNa_V_1.2 determines both isoform specific STX block and isoform specific gating properties.

A stringent test for the possible contribution of the fast inactivated state to phasic TTX block is the effect of mutation-induced disabling of the fast inactivated state on use-dependent block. The replacement of a cluster of three hydrophobic amino acids in the intracellular linker connecting domain III and domain IV, isoleucine, phenylalanine, and methionine (IFM > QQQ), by three hydrophilic glutamine residues had been shown to disable fast inactivation [43,44]. Dumaine and Hartmann [45] investigated the effect of abolishing fast inactivation on the use-dependent block in human heart Na^+^ channels. This mutation did not abolish use-dependent TTX block during pulse trains but increased the amplitude of use-dependent block (the maximum block at the end of the pulse trains with 10 μM TTX was 27% in wild type but 42% in IFM > QQQ channels). Furthermore, the onset of use-dependent block in IFM > QQQ was two to three times slower than in wild type. The time course of post-repolarization block was unchanged by the IFM > QQQ mutation. The voltage-dependence of post-repolarization block was in the range of the voltage-dependence of fast inactivation in wild-type. In IFM > QQQ, however, this voltage dependence was shifted to the range of the voltage-dependence of activation. This suggests that post-repolarization block arises from a kinetic state associated with activation and that fast inactivation limits the number of channels in that state. The authors conclude that the activated state is primarily responsible for the TTX induced use-dependent block and that fast inactivation limits the availability of this state. The authors also note that their interpretation of the data is based on the assumption that the removal of fast inactivation does not alter the activation gating or the slow inactivation of the channel. However, the latter assumption may not be valid as slow inactivation has later been shown to be increased in inactivation-defective human heart Na^+^ channels [46].

In 1996 Conti *et al.* [47] presented a quantitative model of the “trapped ion mechanism” of TTX block first suggested by Salgado *et al.* [32]. In this model the channel can exist in three states, an unbound state, a low affinity blocked state and a high affinity blocked state. In the low affinity blocked state a cation is bound to the outer pore lumen and a TTX molecule is bound to the outer vestibule on the top of the ion, thereby trapping the cation in the channel. Because of electrostatic repulsion between the trapped cation and the toxin, the block is weak. Upon opening of the channel, the trapped ion is allowed to escape through the inner pore thereby relieving the electrostatic repulsion with the toxin resulting in a high affinity block with a low off-rate. The new recruitment of weakly blocked channels produces a transient increase in block until the whole process is dominated by the slow dissociation of the toxin molecules from high affinity binding. This model was able to describe phasic block as observed by Makielski *et al.* [33] without assuming high affinity binding to a specific kinetic state thereby supporting the idea that the TTX binding site remains a stable structure during the gating transitions of the sodium channel. The authors suggest that any correlation of use-dependent block with inactivation may be the consequence of a coupling between these processes.

Using this model the authors found that in Na_V_1.4 channels TTX and STX only differed in their “on”-rate constant while having a similar “off”-rate constant [48]. On the other hand, point mutations in the outer pore of Na_V_1.2 channels expressed in *Xenopus* oocytes influenced mainly the “off”-rates of TTX binding [49].

Rosker *et al.* [50] recently performed a systematic study of the blocking action of TTX and its metabolite 4,9-anhydro-TTX in the following isoforms: Na_V_1.2, Na_V_1.3, Na_V_1.4, Na_V_1.5, Na_V_1.6, Na_V_1.7, and Na_V_1.8. The voltage-dependence of activation was not substantially altered by the toxins. However, both toxins shifted the steady-state inactivation to more negative potentials in Na_V_1.6. Furthermore, this shift was concentration-dependent in Na_V_1.6, while no concentration-dependent shift was observed in Na_V_1.7. TTX also shifted the steady state inactivation curve of Na_V_1.5 and increased the slow recovering fraction of Na_V_1.5 channels, while no effect was observed on slow recovery of Na_V_1.6 channels. The authors concluded that the tested toxins “exert functional properties resembling local anesthetics with respect to their effect on steady-state inactivation of Na_V_1.6.”

In summary use-dependent block by TTX and STX has been shown in a number of different preparations, containing both TTX-sensitive and TTX-resistant Na^+^ channels. However, there is no agreement on whether use-dependent block arises from preferential binding to certain kinetic states of the channel or whether it results from interaction of the toxin with a trapped cation. Furthermore, among those authors suggesting state-dependent binding as mechanism of use-dependent toxin action there is no agreement on the nature of the high affinity kinetic state. The assumption of state-dependent block would imply some conformational change of the outer vestibule. As mentioned in the introduction, there are both functional and structural data supporting the concept of a flexible pore structure. On the other hand, the “trapped ion” model does not require the assumption of a conformational change of the toxin receptor during gating. In the framework of this model, however, any correlation of use-dependent block with inactivation must be the consequence of a tight coupling between activation and inactivation. It appears difficult to reconcile this idea with the recent findings by Rosker *et al.* showing that in Na_V_1.6 TTX did not alter the voltage-dependence of activation but produced a significant shift in the voltage-dependence of inactivation [50]. Perhaps rate-dependent toxin block by TTX results from a complex isoform-specific interaction of several mechanisms. It should be noted that structural data obtained in the KcsA channel suggest that the selectivity filter undergoes conformational changes during permeation [51]. Boccaccio *et al.* proposed that such conformational changes of the selectivity filter, depending on the nature of the bound ion may modulate toxin binding [52]. Such mechanism may provide a link between the “trapped ion” model of TTX binding and state-dependent channel modulation by the toxin.

## 3. Partial Blockers of the Outer Vestibule Allow Assessment of Changes in Gating Behavior during Drug Binding

The investigation of possible alterations of the conformation of the external vestibule by TTX is limited by the fact that channels in the blocked state are non-conducting and any detectable current is flowing through unblocked channels. Nevertheless, during the blocked state it is still possible to measure currents produced by the movement of the voltage-sensors during gating transitions (“gating currents”) [5]. In such experiments performed in crayfish axons STX and TTX were found to induce shifts in the voltage dependence of gating currents associated with fast inactivation [53]. Furthermore, in squid giant axons TTX was found to reduce the size of a late component of the gating current consistent with a structural change induced by the toxin during the final opening step of the channel [54]. However, the assessment of possible actions of TTX on *slow* gating transitions during the blocked state may not generate detectable gating currents. Such potential effects of TTX on slow gating transitions are reasonable as the outer vestibule has been implicated in slow inactivation behavior [55]. Furthermore, slow inactivation in K^+^ channels (“C-type inactivation”) can be modulated by binding of TEA to the outer vestibule [56]. Unfortunately, the dissociation of TTX from the channel is slow which makes it impossible to use ionic currents to assess kinetic changes during the blocked state.

This problem may be circumvented by the use of partial pore blockers which allow a residual flow of ionic currents during the blocked state. μ-conotoxin GIIIA (μ-CTX) is a 22 amino acid peptide toxin that blocks the outer pore of the skeletal muscle Na^+^ channel with high affinity [57]. The binding site of μ-CTX overlaps with the binding site of the guanidinium toxins [58–64]. Binding of native μ-CTX to rNa_V_1.4 channels results in complete block of single channel conductance [65]. Thus, possible kinetic effects associated with block of Na^+^ channels by native μ-CTX cannot be assessed by measurements of ionic currents. However, if the mutant of μ-CTX, R13Q, is bound to the Na^+^ channel outer vestibule, single channel currents during openings are reduced by about 25–30%, indicating partial block of the channel [66]. Hence, the mutant toxin appears to enter the outer vestibule but does not completely occlude the pore. This partial block allows assessment of channel gating kinetics in the toxin-bound state. We applied this strategy to the assessment of a kinetic state induced by a mutation in the selectivity filter of the rNa_V_1.4 channel. rNa_V_1.4 channels carrying the mutation K1237E enter a state of very slow inactivation (“ultra-slow inactivation”) in response to a long depolarization (several minutes). Binding of a saturating concentration μ-CTX R13Q markedly reduced the amplitude of ultra-slow recovery from inactivation by >30% and significantly reduced the time constant of ultra-slow inactivation [67]. This indicates that binding of this toxin to a region which overlaps with the TTX binding site substantially influences channel gating. Furthermore, the ultra-slow inactivated state is not only confined to a conformational change of the outer vestibule but is also linked to activation [68], fast inactivation [69] and binding of local anesthetics [70]. Thus the outer vestibule appears to be part of gating apparatus. This notion is supported by structural data acquired in the KcsA K^+^ channel: Recently, Cuello *et al.* solved the structure the bacterial KcsA channel in different open conformations corresponding to varying states in the activation-inactivation pathway. Analysis of these structures suggested that residue F103 in TM2 interacts with the C-terminal end of the pore helix thereby altering the structure of the outer vestibule. This conformational change gives rise to a non-conductive conformation of the selectivity filter [71].

## 4. Gating Changes by Mutations in the Outer Vestibule—A Topological Overview

Given the possible role of the outer vestibule of the voltage-gated Na^+^ channel in gating transitions the question arises whether such gating changes are physiologically relevant and whether certain residues in the vestibule can be assigned specific roles in channel gating. Therefore, we performed a systematic search for published gating changes associated with mutations in the outer vestibule. We looked both for the effects of engineered mutations and for mutations occurring naturally. We searched the PubMed database (http://www.ncbi.nlm.nih.gov/sites/entrez) for both engineered mutations and spontaneously occurring mutations (channelopathies) in all isoforms of Na^+^ channels. Only those mutations that produce kinetic changes were considered. We excluded mutations that were not investigated electrophysiologically or were found during electrophysiologic studies not to generate current. We only looked for mutations in the outer channel vestibule as defined in the Lipkind-Fozzard homology model [14] that is based on the crystal structure of the bacterial KcsA channel [72]. If for a given site data for replacements by different amino acids were available we only considered those with the greatest effect. Table 1 gives an schematic overview of the retrieved data. The amino acid codes in the rows correspond to the rNa_V_1.4 sequence although the indicated mutations do not have to be reported in this isoform. In order to keep the information in the table condensed it was necessary to make a number of generalizations and arbitrary definitions. Color codes are used instead of numbers to allow for a better topologic analysis. Grey boxes indicate the residues of the selectivity filter (DEKA locus). All mutations were engineered with the exception of those indicated by yellow boxes which represent naturally occurring mutations (channelopathies). All mutational effects on kinetic states were classified as “no change” (green), as “enhancement” (red) or “reduction” (blue). Furthermore, we classified the data in a semi-quantitative way as minor or major effect (light *versus* dark coloring, respectively). Kinetic changes in activation were judged by their effect on the half point of the conductance *versus* voltage curve. Negative shifts were classified as “enhancement”, positive shifts as “reduction”. Shifts were considered “minor” if they were in the range of 2–5 mV. Shifts > 5 mV were considered “major”. In case of the inactivated state a number of generalizations were made:

1. Fast and slow inactivated states were lumped together.
2. Effects on the half point of steady state availability were categorized as described for the conductance *versus* voltage curve. Steady state probability of an inactivated state was considered “major” if the change was >50%. Effects on time constants of development and/or recovery were considered “minor” if the change was 1.5–2 fold. >2-fold changes were considered “major”. The same criteria were applied to changes in the relative amplitude of the respective state(s).
3. If changes in several parameters were reported for a given state, and/or if several inactivated states were reported we considered the respective value/state with the greatest change.

If the values of peak current in the mutant were 20–50% of wild-type, the change was considered minor, values < 20% of wild type were classified as major reduction.

**Figure 2:**
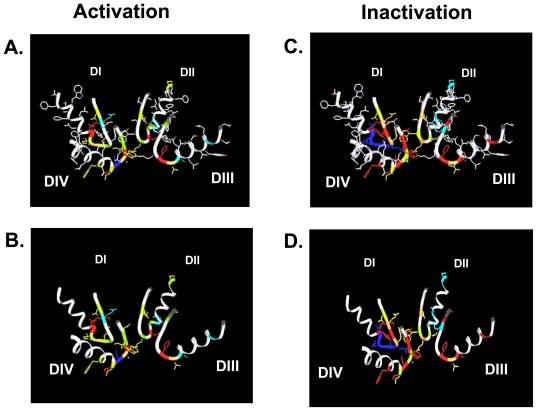
The gating changes presented in Table 1 are indicated in the Lipkind Fozzard model of the outer vestibule of the voltage-gated Na^+^ channel [92]. Shown are the P-loops of all four domains (DI-DIV). In panels A. and C. all amino acid side chains are depicted. In panels B. and D. only the side chains of amino acid positions for which gating changes are reported in Table 1 are shown. A. and B. present changes in activation, C. and D. show changes in inactivation. The color code corresponds to Table 1.

Table 1 and Figure 2 show that most mutations in the P-loop region are associated with changes in gating. Interestingly, mutations in the P-loop of domain I seem to decrease inactivation while mutations in the P-loops of domain IV appear to enhance inactivation. In this regard it should be noted that the voltage sensor of domain IV has been implicated in inactivation gating [93–98]. Also, in the channel structure the P-loops of domains I and IV are in close proximity to each other (Figure 2) [14]. Furthermore, the P-loop of domain IV is predicted to be in close proximity to the domain IV S6 segment [14] which also has also a prominent role in inactivation [99–105] and contains the binding sites for local anesthetics [106,107]. Binding of local anesthetics is influenced by amino acids in the P-loops [108] and local anesthetic binding has been shown to alter the conformation of the outer vestibule [109]. Perhaps P-loops and adjacent S6 segments form a gating trajectory as has been proposed for K^+^ channels [110,111].

Table 1 and Figure 2 also show that in domain I amino acid changes immediately N-terminal of the selectivity filter (D400) decrease inactivation, while mutations C-terminal of the putative selectivity filter increase inactivation. This is notable as position 401, immediately C-terminal to the selectivity filter residue D400, has been shown to determine TTX sensitivity/resistance [41]. Of special interest is residue site 754 in the P-loop of domain II of the human skeletal muscle Na^+^ channel (hNa_V_1.4, corresponding to V748 in rNa_V_1.4). Vilin *et al.* showed that the amino acid at this site determines the isoform specific property of slow inactivation in skeletal and heart Na^+^ channels. Thus, substituting V754 in hNa_V_1.4 (skeletal muscle) with isoleucine from the corresponding position (891) in hNa_V_1.5 (heart) reduced the amount of steady-state slow inactivation to the level found in wild-type hNa_V_1.5 channels.

## 5. Physiological Relevance of Gating Changes by Mutations in the Outer Vestibule

So far we have considered the involvement of outer vestibule of the voltage-gated Na^+^ channel in channel gating from a pure biophysical standpoint. However, a number of amino acid positions listed in Table 1 have important pathophysiological consequences. As mentioned above position 754 in hNa_V_1.4 (skeletal muscle) and 891 in hNa_V_1.5 (heart) determine the isoform specific probability of entry into slow inactivation. It has been proposed that the high probability of slow inactivation of hNa_V_1.4 channels has a physiological role in muscle fatigue [112]. On the other hand, the low probability of slow inactivation in hNa_V_1.5 (heart) may prevent the potential rundown of cardiac muscle excitability during the long duration of the cardiac action potential and during repetitive contractions [75].

Mutations in genes encoding for voltage-gated Na^+^ channels have long been recognized to give rise to various diseases (“channelopathies”) of skeletal and heart muscle as well as the central and peripheral nervous system (for a review, see [113]). The yellow boxes in Table 1 indicate those reports of human channelopathies associated with mutations in the P-loops, in which channel gating was assessed. Not considered are mutations in the outer vestibule that completely abolish channel function. The majority of the listed mutations give rise to Brugada syndrome, a clinical entity characterized by a specific ECG pattern and an increased risk for potentially lethal ventricular arrhythmias [114]. The syndrome is thought to account for up to 4% of all sudden cardiac deaths and 20% of unexplained sudden death in the setting of a structurally normal heart [115]. Presently, there are reports of over 100 mutations associated with Brugada syndrome (http://www.fsm.it/cardmoc) [115]. Most mutations associated with Brugada syndrome produce a loss of function of the cardiac Na^+^ channel [114]. These mutations can be found in all parts of the channel protein [115]. Reported mutations in the P-loops that result in gating alterations giving rise to Brugada syndrome are: G1406R (domain III) [74], D1714G (domain IV) [90], S1710L (domain IV) [86,116]. Interestingly, the only gating alteration which is common to all reports is an enhancement of slow inactivation, which underscores the importance of the outer vestibule in the modulation of this kinetic state. Apart from Brugada syndrome, gating changes produced by a mutation in the outer vestibule have been found in a case sudden infant death syndrome (SIDS) [76]. This syndrome is defined as the sudden unexpected death of an infant <1 year of age, with onset of the fatal episode apparently occurring during sleep, which remains unexplained after thorough investigation [117]. Otagiri *et al.* reported that the SIDS associated mutation F1705S in the domain IV P-loop of the cardiac sodium channel gave rise to changes in the slope factor of the voltage dependence of activation and to a hyperpolarizing shift of the voltage dependence of steady-state inactivation [76]. Furthermore, the time constants of recovery from fast and from slow inactivation were increased by the mutation, again implicating the outer vestibule in the control of this kinetic state.

Apart from human disease, gating changes produced by mutations in the outer vestibule may have broad biological implications. Thus, natural mutations conferring TTX-resistance to animals are sometimes associated with functional changes resulting in impairment of motor function which may influence the locomotive performance (for review see [9]). Thus, the study of the delicate balance between benefits and costs of TTX-resistance in the animal kingdom may lead to intriguing new insights into evolutionary mechanisms.

## 6. Conclusions

While in K^+^ channels the role of outer vestibule as a gating structure is a well established (for review see [118]), the role of the outer pore in voltage-gated Na^+^ channels in the channel’s gating behavior is less well understood. Nevertheless, considering (1) the recent structural information from K^+^ channels suggesting a high degree of conformation flexibility of the P-loop region, (2) the complex data regarding use-dependent block by guanidinium toxins and (3) the reported kinetic changes by site-directed mutagenesis in the outer vestibule, it appears resonable to assume that gating transitions in this region may be involved in the interaction between guanidinium toxins and the outer vestibule of voltage-gated Na^+^ channels.

## Figures and Tables

**Table 1:**
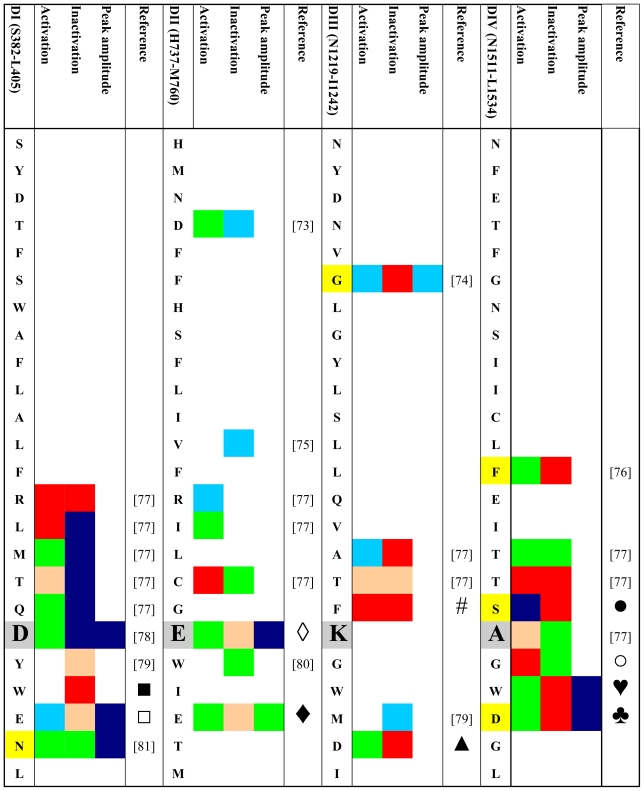
Reported effects of mutations in the P-loop region on selected gating parameters and peak inward currents.

The symbols in the table link to the following publications: ▪ = [80,82,83]; □ = [79,84,10,85]; ⋄ = [73,77]; ♦ = [73,84,85]; ▴ = [85,84]; ● = [77,86,87,116]; ○ = [79,80,88]; &hearts; = [88,89]; ♣ = [84,90]; # = [77,91].

Color code: Yellow: naturally occurring mutations (channelopathies); Grey/large letters: putative selectivity filter; Green: no change; Light red: minor enhancement; Dark red: major enhancement; Light blue: minor reduction; Dark blue: major reduction; See text for further information.
